# Reproducibility of aortic pulse wave velocity measurements obtained with Phase Contrast Magnetic Resonance (PCMR) and applanation tonometry

**DOI:** 10.1186/1532-429X-11-S1-P228

**Published:** 2009-01-28

**Authors:** Jonathan Suever, David Huneycutt, Enrique Rojas-Campos, Francesca Cardarelli, Amol Pedneker, Sam Fielden, Arthur Stillman, Paolo Raggi, John Oshinski

**Affiliations:** 1 Biomedical Engineering, Emory University/Georgia Institute of Technology, Atlanta, GA USA; 2 Radiology, Emory University School of Medicine, Atlanta, GA USA; 3 Cardiology, Emory University School of Medicine, Atlanta, GA USA; 4 Philips Medical Systems, Cleveland, OH USA

**Keywords:** Pulse Wave Velocity, Applanation Tonometry, Aortic Pulse Wave Velocity, Phase Contrast Magnetic Resonance, Aortic Compliance

## Objective

To compare reproducibility of aortic pulse wave velocity (PWV) measurements obtained from phase contrast magnetic resonance (PCMR) and cross-correlation analysis to applanation tonometry.

## Background

Increased pulse wave velocity (PWV) results from arterial stiffening and is commonly seen with ageing and in patients with atherosclerosis and/or hypertension [1]. Currently, applanation tonometry is the clinically accepted method to quantify aortic PWV and determine aortic compliance. A direct comparison of PWV values and reproducibility between PCMR and tonometry has not been done.

## Methods

PWV was measured in ten healthy volunteers (age: 32.9 ± 8.3 years) two times with each modality. To obtain PCMR measurements, subjects were scanned in a 1.5 T Philips Intera CV scanner (Philips Medical Systems, Best, The Netherlands). Oblique sagittal images in the plane of the aorta were acquired and velocity was encoded in the foot-head direction. After the first PCMR scan, subjects were removed from the scanner, repositioned, and the scan was repeated. After two PCMR scans, subjects remained in the supine position and underwent two measurements of the aortic (carotid-to-femoral) PWV by applanation tonometry using a Sphygmocor device (AtCor Medical, Sydney, Australia) in an adjacent room.

PCMR data were analyzed by examining the velocity waveforms at multiple points along the length of the thoracic and abdominal aorta throughout the cardiac cycle. Cross correlation between flow waveforms was then used to determine the transit time between two adjacent locations along the aorta [2]. A robust bisquare-weighted linear regression algorithm was used to fit a line to the arrival time/location plot, which was then used to compute PWV. Applanation tonometry data were analyzed automatically by the Sphygmocor software using a standard transfer formula.

Agreement and repeatability of PCMR and tonometry measurements were assessed by Bland-Altman statistics [3]. Inter-scan variability for each modality was evaluated with the Coefficient of Variation (CV). P-values less than 0.05 were considered statistically significant.

## Results

**Figure 1:**
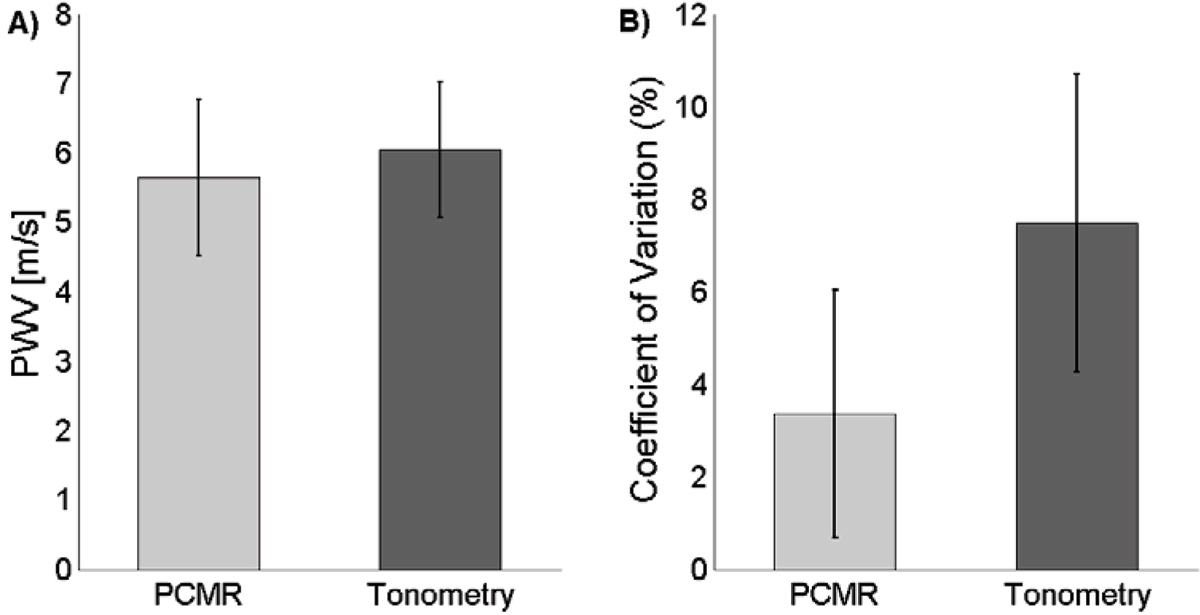
**a) Average PWV for all subjects for both PCMR and tonometry (P > 0.05)**. b) Inter-scan coefficient of variation for each modality (P < 0.05).

Mean PWV values from PCMR and applanation tonometry were not statistically different (PCMR: 5.6 ± 1.1 m/s; Tonometry: 6.0 ± 1.0, P > 0.05) (Figure 1a). The average coefficient of variation (CV) was significantly lower for PCMR compared to tonometry, 3.4% ± 2.7% versus 7.5% ± 3.2%, respectively (P < 0.05) (Figure 1b). Bland Altman analysis showed that there was a 0.4 m/s bias with tonometry being higher and a confidence interval of ± 1.3 m/s.

## Conclusion

PWV was successfully measured by PCMR combined with cross-correlation analysis and by applanation tonometry. There was no significant difference in the PWV values; however, PCMR has a statistically significant lower inter-test CV compared to tonometry. It can be concluded that PCMR and cross-correlation analysis can be combined to accurately assess PWV as an estimate of aortic compliance.
